# Genetic fine-mapping of *DIPLOSPOROUS *in *Taraxacum *(dandelion; Asteraceae) indicates a duplicated *DIP*-gene

**DOI:** 10.1186/1471-2229-10-154

**Published:** 2010-07-26

**Authors:** Kitty Vijverberg, Slavica Milanovic-Ivanovic, Tanja Bakx-Schotman, Peter J van Dijk

**Affiliations:** 1 Netherlands Institute of Ecology (NIOO-KNAW), Centre for Terrestrial Ecology, NL 6666GA Heteren, The Netherlands; 2 Current Address: Plant Genetics, IWWR, Radboud University Nijmegen, Heyendaalseweg 135, NL-6525AJ Nijmegen, The Netherlands; 3 Current Address: Keygene NV, NL-6708PW Wageningen, The Netherlands

## Abstract

**Background:**

*DIPLOSPOROUS *(*DIP*) is the locus for diplospory in *Taraxacum*, associated to unreduced female gamete formation in apomicts. Apomicts reproduce clonally through seeds, including apomeiosis, parthenogenesis, and autonomous or pseudogamous endosperm formation. In *Taraxacum*, diplospory results in first division restitution (FDR) nuclei, and inherits as a dominant, monogenic trait, independent from the other apomixis elements. A preliminary genetic linkage map indicated that the *DIP*-locus lacks suppression of recombination, which is unique among all other map-based cloning efforts of apomeiosis to date. FDR as well as apomixis as a whole are of interest in plant breeding, allowing for polyploidization and fixation of hybrid vigor, respectively. No dominant FDR or apomixis genes have yet been isolated. Here, we zoom-in to the *DIP*-locus by largely extending our initial mapping population, and by analyzing (local) suppression of recombination and allele sequence divergence (ASD).

**Results:**

We identified 24 recombinants between two most closely linked molecular markers to *DIP *in an F1-population of 2227 plants that segregates for diplospory and lacks parthenogenesis. Both markers segregated c. 1:1 in the entire population, indicating a 1:1 segregation rate of diplospory. Fine-mapping showed three amplified fragment length polymorphisms (AFLPs) closest to *DIP *at 0.2 cM at one flank and a single AFLP at 0.4 cM at the other flank. Our data lacked strong evidence for ASD at marker regions close to *DIP*. An unexpected bias towards diplosporous plants among the recombinants (20 out of 24) was found. One third of these diplosporous recombinants showed incomplete penetrance of 50-85% diplospory.

**Conclusions:**

Our data give interesting new insights into the structure of the diplospory locus in *Taraxacum*. We postulate a locus with a minimum of two *DIP*-genes and possibly including one or two enhancers or *cis-*regulatory elements on the basis of the bias towards diplosporous recombinants and incomplete penetrance of diplospory in some of them. We define the *DIP*-locus to 0.6 cM, which is estimated to cover ~200-300 Kb, with the closest marker at 0.2 cM. Our results confirm the minor role of suppression of recombination and ASD around *DIP*, making it an excellent candidate to isolate via a chromosome-walking approach.

## Background

Most seed plants reproduce sexually, but a proportion of c. 0.1% reproduce asexually through seeds, a process also known as apomixis, *sensu stricto*, or agamospermy [1,2]. In apomictic plants, the embryo sac arises from an *unreduced *gametophytic cell (diplospory) or a sporophytic cell of the ovule (apospory), followed by further gametogenesis. At maturity, the embryo develops parthenogenetically (without fertilization) from the egg cell, and the endosperm develops pseudogamously (requiring fertilization) or, in a minority of apomictic species, autonomously, from the central cell [reviewed by [3]]. Diplosporous apomixis can be mitotic, that is, without meiosis at all, or meiotic, in which meiosis is entered but results in nuclear restitution [4,5], also known as first division restitution (FDR [6]). Apart from possible mutations, apomictic offspring are genetically identical to the mother plant. Apomixis is restricted to the female reproductive pathway, giving the opportunity to investigate its genetics in hermaphroditic apomicts by using them as pollen donors. With the exception of *Allium *and some (sub)tropical fruit trees and forage grasses, natural apomixis is absent from crop species.

Apomixis is of interest in plant breeding and seed production, because it allows for the fixation and unlimited propagation of complex and heterozygous genotypes [7]. Engineering apomixis into crop plants could promise social and economic benefits that would challenge those of the Green Revolution [8]. Advantages of apomictic reproduction also include the rapid generation of superior forms from novel germplasm, the avoidance of complications associated with sexual reproduction, e.g., self-incompatibility, and the prevention of horizontal disease transfer [2,9]. Separate elements of apomixis can be of interest in plant breeding in various ways, such as for raising the ploidy levels by using diplospory or apospory, forming maternal dihaploids by using parthenogenesis, and alternating ploidy levels between generations by applying these elements alternatively [10]. In a more fundamental sense, apomixes is of interest in the context of the paradox of sex, questioning why sexual reproducing organisms are so successful in nature despite advantages of asexual reproduction [11-13]. For all of these interests, the genetics and molecular basis of apomixis have been investigated intensively during last decades [reviewed by [2,14]]. Despite these efforts, the understanding of the molecular basis of apomictic reproduction and its use as a tool in plant breeding are still at their beginning.

Efforts to understand the molecular basis of apomixis and to introduce it into crop species include the introgression via *classical breeding *approaches with apomictic relatives [reviewed by [15]], the unraveling of its genetic control in *natural *apomicts followed by attempts to map-based clone the genes associated [reviewed by [16]], and the identification of genes that control elements of apomixis in well-defined *sexual *species [reviewed by [17]]. Up to now, stable introgression of apomixis into crops via breeding, together with an accompanying preservation of seed yield, has failed. The isolation of apomixis genes from natural apomicts appeared also be difficult, because in most species these genes are associated with large genomic regions in which recombination is suppressed. Despite this difficulty, evidence increases for the multigenic control of apomixis, particularly the separate control of apomeiosis and parthenogenesis [16,18,19], and genes associated with apospory have been reported, including *BABY BOOM-like *genes in *Pennisetum *[20] and *HAPPY *in *Hypericum *[19]. Some recent progress has been made with the third approach in the model species *Arabidopsis*, by identifying the *DYAD/SWITCH*1 (*SWI*1) gene [21] and characterizing *dyad *plants [22], and by identifying the *Omission of Second Division*1 (*OSD*1) gene and creating *osd*1/*Atspo*11-1/*Atrec*8 triple mutants (*MiMe*) [23]. *DYAD/SWI*1 is a regulator of meiotic chromosome organization and mutation of this gene leads to FDR. *OSD*1 mutants result in SDR, and in the triple mutant form *MiMe*, to mitotic division. Although *dyad *is recessive and results in the production of unreduced gametes at low frequencies and *MiMe *is a triple mutant, these findings are first steps in the possible engineering of apomixis or elements thereof in plant breeding. A third gene that was recently found to be involved in female gamete formation is *ARGONAUTE*9 (*AGO*9) [24]. Mutations of this gene lead to developing megagametophytes also from somatic cells, being reminiscent of apospory. Here, we focus on the second approach, using the natural apomict *Taraxacum officinale *(common dandelion, Asteraceae) in order to map diplospory.

Attempts to map apomeiosis, either diplospory or apospory, have particularly been made in species of the Poaceae (grasses) and Asteraceae [reviewed by [16,25]]. The general view emerging from the studies in Poaceae is that apomixis inherits as a whole as a dominant, monogenic trait, and that molecular markers cluster with the trait, indicating (strong) suppression of recombination at these loci. This was found for aposporous species of *Panicum *[26], *Paspalum *[27,28], *Pennisetum *and *Cenchrus *[29-31], and diplosporous *Tripsacum dactyloides *[32]. Exceptions were found in *Brachiaria*, that lacked clustering of markers at the locus for aposporous apomixis in an initial mapping population [33], and in *Poa*, that showed independent inheritance of apospory and parthenogenesis and no marker-clustering at the locus of the latter, whereas apospory was not mapped [34]. In a later study in *Poa*, a five locus model for the inheritance of apomixis confirmed the independent inheritance of apospory and parthenogenesis [35]. A recent study in *Panicum *indicated independent inheritance of all three apomixis elements in this species [18]. The general view from studies in Asteraceae is that apomeiosis and parthenogenesis inherit independently and that suppression of recombination is indicated at some but not all loci. In *Erigeron annuus*, the locus for diplospory showed clustering of markers, whereas the locus for parthenogenesis did not [36]. In *Hieracium *spp., a deletion mapping study was initiated in order to avoid anticipated difficulties associated with map-based cloning at recombinationally suppressed loci [37]. The results showed independent loci for apospory and parthenogenesis, designated *Loss Of Apospory *(*LOA*) and *Loss Of Parthenogenesis *(*LOP*), respectively. In *Taraxacum*, a mapping study showed regular distribution of markers over the *DIPLOSPOROUS *(*DIP*)-chromosomal region, indicating the occurrence of recombination in this region [38]. Preliminary mapping of *PARTHENOGENESIS *(*PAR*) in this species showed co-segregation of markers with the trait, suggesting suppression of recombination at this locus [PJ Van Dijk *et al. *unpublished results]. Since the *DIP*-chromosomal region in *Taraxacum *lacks evidence for suppression of recombination, it is one of the best candidates to isolate (a) gene(s) for apomeiosis via a map-based cloning approach.

The suppression of recombination found in most mapping studies of apomixis loci to date indicate (strong) divergence of alleles at these loci. Evidence for this was particularly found in *Pennisetum *and *Paspalum *that showed hemizygosity at the loci for aposporous apomixis [29,28]. Allele sequence divergence (ASD) can be expected under (long-term) asexual, non-recombining, conditions [39]. Depending on the time of asexuality, the two (or more) alleles of a gene can acquire high levels of heterozygosity and the homologous chromosomes can accumulate different chromosome rearrangements. Experimental evidence for ASD comes from the *Bdelloid rotifers*, an ancient asexual lineage of freshwater invertebrates, showing greater sequence divergence between formerly alleles of several protein-coding genes in a single individual than between the same loci in related species [40,41]. In hermaphroditic apomicts, in which the male sexual pathway allows for recombination, the process of ASD will be retarded, but will, according to computer simulations, still occur [42]. ASD can also be reduced by gene conversion and mitotic crossing-over. In *T. officinale*, no evidence for suppression of recombination was found at the *DIP-*locus [38]. At one flank at ~3.5 cM from *DIP*, linkage at 0-1 cM was found between two microsatellite loci (Mst53 and Mst78) that were 5-10 cM apart in sexual relatives [42-44]. This might refer to local suppression of recombination at a region close to *DIP *and was among our investigations of the study presented here.

*T. officinale s.l*. consists of polyploid, mostly triploid (*2n *= *3x *= *24*) apomicts and diploid (*2n *= *2x *= *16*) sexual individuals [45]. Apomictic *Taraxacum *is diplosporous, with parthenogenetic embryo development and autonomous endosperm formation [46]. Diplospory is of the meiotic type (*Taraxacum *type [5]) in which bivalent formation during female meiosis I is omitted or occurs at low frequencies [47-50], followed by nuclear restitution and normal meiosis II. Molecular marker studies lacked evidence for genetic exchange during this process, but more extensive studies on genetic consequences of possible recombination events have to be performed. Crossing studies revealed that diplospory and parthenogenesis inherit independently in *T. officinale *[51], whereas the precise inheritance of autonomous endosperm formation is yet unresolved. Diplospory showed dominant, monogenic inheritance, indicating genotypes *Ddd*, *Dddd*, and *dd *for tri- and tetraploid apomicts and diploid sexuals, respectively [44]. Parthenogenesis also inherits as a dominant, monogenic trait [[51], PJ Van Dijk *et al. *unpublished results]. A significant linkage of the rDNA locus to two Mst loci, Mst53 and Mst78, that were linked to diplospory, suggest a physical location of *DIP *on one of the nucleolar organizer region (NOR) chromosomes [44]. This was in agreement with a cytological observation in a triploid disomic plant (2*n *= 3*x *= 24-1) that lacked one of the NOR-chromosomes accompanying with the loss of apomixis [52,44]. Fluorescent *in situ *hybridization (FISH) experiments with bacterial artificial chromosomes (BACs) linked to *DIP *also indicated a location of *DIP *on one of the NOR-chromosomes [RJ Vašut *et al. *unpublished results]. Apomixis in *Taraxacum *is mostly obligate, indicating that the dominant *DIP *allele is fully penetrant. Occasional formation of quite a high number of bivalents followed by meiotic reduction has, however, been reported [49] as were crossing experiments that resulted in some progeny that indicated reduced egg cells from the diplosporous seed parent [53]. These observations may indicate that *DIP *is not fully penetrant in *Taraxacum *in all accessions or under all conditions. Since *DIP *is expressed during female meiosis only, it is transferred via reductional and recombinational male meiosis, thereby allowing us to map the trait.

A first map of *DIP *was based on 73 plants of a segregating population, 34 amplified fragment length polymorphisms (AFLPs) resolved by a bulked segregant analysis and linked alleles at two Mst loci [38]. Total length of this map was 18.6 cM and markers were found at both sides of *DIP*, corresponding to 5.9 and 12.7 cM, respectively. None of the markers completely co-segregated with *DIP*, and the closest markers concerned unresolved groups of three AFLPs at 1.4 cM from *DIP *at each flank. The regular distribution of markers over the *DIP*-chromosomal region indicated that extensive suppression of recombination was lacking at the apomeiosis locus in *T. officinale*. A number of AFLPs was successfully converted into PCR-based markers in order to facilitate further fine mapping of *DIP *as well as to compare sequences of marker regions between *DIP *and non-*DIP *homolog's [38]. The results included two dominant sequence characterized amplified regions (SCARs) from within the AFLP-groups closest to *DIP*: S8 and S10, and one co-dominant single nucleotide polymorphism (SNP) at a larger distance from *DIP*: S4. The other markers showed PCR-products in sexual as well as apomictic individuals, lacking evidence for obvious sequence divergence between the *DIP *and non-*DIP *homolog's or the presence of hemizygosity at the *DIP*-chromosomal region in *Taraxacum*.

As part of our map-based cloning effort of *DIP*, we here continued the genetic linkage mapping of *DIP *and the characterization of the *DIP*-chromosomal region in *T. officinale*. We extended the segregating population used for initially mapping to a total of 2227 plants. This population included diplospory in the paternal plant, but lacked parthenogenesis. Plants were screened for recombination between the PCR-markers S8 and S10, and recombinants were phenotyped for their mode of reproduction and analyzed for AFLPs known to be closely linked to *DIP*. Since the *DIP *locus is exceptional among apomixis loci in that it lacks evidence for suppression of recombination, we investigated the occurrence of recombination in a larger region around *DIP *in more detail. We analyzed a random selection of 300 plants for S4, Mst78a and Mst53b in addition to S8 and S10, and compared the distance between the Mst loci with those between alleles at non-*DIP *homolog's. Allele sequence divergence between the *DIP *chromosome and non-*DIP *homolog's was measured at six marker regions at different distances from *DIP*. For this, we sequenced cloned PCR-products originating from the sexual parent, a diplosporous offspring plant, and an unrelated apomictic dandelion. Our results resolved a more detailed map of *DIP *and confirmed that suppression of recombination and allele sequence divergence play a minor role in the *DIP*-chromosomal region in *T. officinale*. We found an unexpected bias towards diplosporous reproducing recombinants, which we explained by postulating a duplicated *DIP*-locus rather than a single gene.

## Results

### Fine-mapping of *DIPLOSPOROUS*

**Figure 1:**
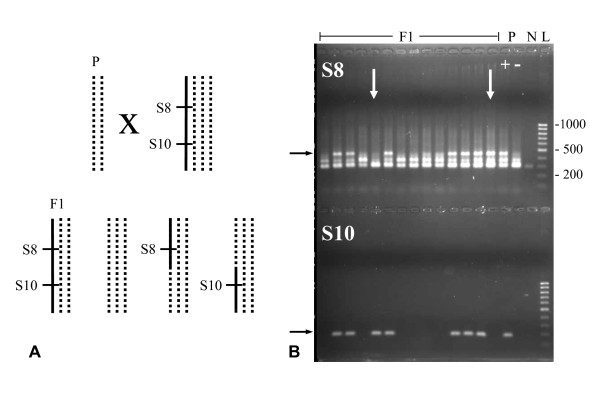
**Genotypes of parents (P) and offspring (F1) of the cross used to map diplospory in *T. officinale***. **(A) **Crossing scheme showing the sexual diploid maternal plant S2.125 (*dd*, dotted lines), the diplosporous tetraploid pollen donor PAX (*Dddd*, with *D *= solid line), and their possible triploid offspring (*Ddd*, *ddd*, and *D/d*-*dd *recombinants). S8 and S10 span *DIP *in 2.7 cM [38], and the S8+/S10+ genotypes represent the *D*-alleles. **(B) **Agarose gel showing amplicons of S8 (upper half) and S10 (lower half) of 14 F1 and the two parent plants, with the diagnostic products (black arrows) and recombinant plants (white arrows) indicated. Plants with both markers present were interpreted as being diplosporous and those with both markers absent as being meiotic. N = negative control and L = l00 bp ladder.

In this study, we fine-mapped diplospory in *Taraxacum *by extending the population of 73 plants used for initial mapping [38] to a total of 2227 offspring plants analyzed here. This population originated from a cross between a sexual maternal plant and a diplosporous, but non-parthenogenetic, paternal plant. The dominant SCARs S8 and S10 were used to screen for recombinants. Presence of the diagnostic PCR products of both, S8 and S10, indicated the presence of the *DIP*-chromosomal region, whereas the presence of one of the two markers only indicated a recombination within this region (Figure 1). The results showed 1068 plants (47.9%; Table 1) positive for S8 and S10, 1135 plants (51.0%) negative for both markers, and 24 plants (1.1%) recombinant. From the recombinants, nine were positive for S8 and negative for S10 and 15 showed the opposite score. This made the total frequencies of S8 and S10 fragments 48,4% and 48,6%, respectively, both lacking a significant deviation from the expected 1:1 segregation rate (*S8+/S8- *= 1077:1150, Chi^2 ^= 2.39, p > 0.1; *S10+/S10- *= 1083:1144, Chi^2 ^= 1.67, p > 0.1). Our results confirmed the dominant inheritance of S8+/S10+, respectively the *DIP*-chromosomal region, in a much larger population than screened before. In addition, they reduced the distance between S8 and S10 from 2.7 cM in the previous study to 1.1 cM found here, and indicated that S10 was more closely linked to *DIP *than S8.

**Table 1:** Genotypes and phenotypes of the 2227 plants used to map diplospory

Genotype or Phenotype	# of Plants		**Frequency (%)**^**a**^
S8+/S10+	1068		47.9
S8+/S10- S8-/S10+	9 15	Recs^b^	1.1
S8-/S10-	1135		51.0

S8+	1077		48.4 *^ns^*
S10+	1083		48.6 *^ns^*

*DIP *	1088		48.9 *^ns^*
*MEI *	1139		51.1 *^ns^*

*DIP *Recs^b ^	20		83.3 **
*MEI *Recs	4		16.7 **

^a ^With deviations from a 1:1 segregation rate indicated:

*ns *= p > 0.1; ** = p < 0.01

^b ^Recs = Recombinants

The 24 recombinants between S8 and S10 were phenotyped for their mode of reproduction via crosses with sexual diploid pollen donors followed by analyzing the offspring for their ploidy levels. A total of 20 offspring plants, ten of each of two crosses, were analyzed per recombinant, and chromosome numbers were deduced from flow cytometric analysis (see Methods for exact measurements and definitions). The results showed 20 recombinants (83%; Table 1) to be diplosporous and four being meiotic, clearly deviating from the expected 1:1 segregation rate (*DIP*:*MEI *= 20:4, Chi^2 ^= 10.67, 0.01 > p > 0.001). Fourteen of the diplosporous recombinants showed strong penetrance of diplospory, that is, ≥ of the offspring plants (18 to 20 out of the 20 measured) showed DNA ratios ≥ 1.79 as compared to a diploid (~tetraploid). The other six showed reduced penetrance of diplospory varying from 80% (n = 2), to 75, 70, 63 and 50%. This was supported by two to four additional crosses for each of these recombinant plants (data not shown).

A random set of 61 plants of the mapping population were phenotyped via crosses in an earlier study [44]. This showed 27 diplosporous and 34 meiotic plants, supporting a 1:1 segregation rate (*DIP*:*MEI *= 27:34, Chi^2 ^= 0.80, p > 0.1), and a high penetrance of diplospory in the diplosporous individuals: 98.5% of the offspring showed the tetraploid chromosome set. The *DIP*-linked AFLPs co-segregated with the diplosporous phenotype [38]. Accordingly, we deduced the diplosporous phenotype and a high penetrance of diplospory from the S8+/S10+ genotype in the remainder of the population. The final result showed 1088 diplosporous (1068 S8+/S10+ plants plus 20 diplosporous recombinants = 48.9%; Table 1) and 1139 meiotic plants (51,1%), confirming the expected 1:1 segregation rate (*DIP*:*MEI *= 1088:1139, Chi^2 ^= 1.17, p > 0.1). In summary, our results showed a strong bias towards diplosporous individuals among the recombinants, whereas they confirmed a 1:1 segregation rate of diplospory *versus *meiotic reproduction in the entire mapping population. One third of the diplosporous recombinants showed incomplete penetrance of diplospory.

Further fine-mapping of *DIP *was based on the 24 recombinants and the AFLPs known to be closely linked to *DIP *[38]. One of the two most closely linked clusters of three AFLPs showed resolution: S7 was closer to *DIP *than S8 and the third marker of this group. The other cluster remained unresolved, indicating that the markers of this group are as far or farther from *DIP *than S10 on the genetic map. The markers at larger distances from *DIP *co-segregated with the more closely linked AFLPs as expected, confirming the nature of the recombinants as well as the results from the previous study. Five recombinants showed a recombination between *DIP *and S10 and 19 between *DIP *and S8 (Table 2), supporting the above made suggestion that S10 is closer to *DIP *than S8. Our results reduced the map length of the *DIP*-locus from 1.1 cM between S8 and S10 to 0.6 cM between S7 and S10. They showed that S7 was most closely linked to *DIP *at one flank, at 0.4 cM, and S10 probably at the other flank, at 0.2 cM.

**Table 2:** Recombination frequencies and distances between markers at the *DIPLOPOROUS*-chromosomal region in *T. officinale*

Map	A		B		C	
	#	length (cM)	#	length (cM)	#	length (cM)
Population Size	73		300		2227	
						
Recombinants						
S4 - Mst53b	7	9.6 ± 6.8^a^	22	7.3 ± 3.0	nt^b^	-
S4 - S8	2	2.7 ± 3.7	2	0.7 ± 0.9	nt	-
S10 - Mst78a	3	4.1 ± 4.6	10	3.3 ± 2.0	nt	-
Mst78a - Mst53b	0	0	7	2.3 ± 1.7	nt	-
						
S8 - S10	2	2.7 ± 3.7	3	1.0 ± 1.1	24	1.1 ± 0.4
S8 - *DIP*	1	1.4 ± 2.7	1-2	~0.5 ± 0.8	19	0.9 ± 0.4
*DIP *- S10	1	1.4 ± 2.7	1-2	~0.5 ± 0.8	5	0.2 ± 0.2
						
S7 - S10	2	2.7 ± 3.7	nt^b^	-	14	0.6 ± 0.3
S7 - *DIP*	1	1.4 ± 2.7	nt	-	9	0.4 ± 0.3
*DIP *- S10	1	1.4 ± 2.7	1-2	~0.5 ± 0.8	5	0.2 ± 0.2

^a ^Confidence intervals were calculated according to Wu *et al. *[71], see Methods

^b ^nt = not tested

The marker distribution over a larger *DIP*-chromosomal region was investigated on the basis of 300 individuals, including the 73 plants of the initial mapping population and a random set of 227 plants chosen from the extended population used here. These plants were screened for the PCR-markers S4, Mst78a and Mst53b in addition to S8 and S10. Results are summarized in Table 2. The distance between the most widely separated markers, S4 and Mst53b, was 7.3 ± 3.0 cM, which was comparable to 9.6 cM found previously [38]. The distance between Mst78a and Mst53b was 2.3 ± 1.7 cM, which was in a better agreement with the 5-10 cM found at non-*DIP *homolog's in *Taraxacum *in earlier studies [42-44] than their close linkage in our initial *DIP*-map [38]. It also non-significantly deviated from the 6.3 cM between these Mst alleles at the non-*DIP *homolog's in the diploid sexual parent S2.125 in this study (Chi^2 ^= 1.86, p > 0.1). The distance between the Mst78 and Mst53 alleles at non-*DIP *homolog's in the tetraploid diplosporous parent PAX varied from 4.7 cM to 10.3 and 12.7 cM, the latter two significantly deviating from the 2.3 cM found at the *DIP*-homolog. Excluding the results from one of the five flower heads, because it showed a strong bias towards recombination's between two particular allele combinations, reduced the distance between the Mst alleles to 2.0 cM at the *DIP*-chromosome and 3.7, 4.3 and 6.7 cM at the non-*DIP *homolog's in PAX, lacking a significant deviation between the extremes (Chi^2 ^= 2.54, p > 0.1). In summary, our results confirmed the occurrence of recombination in the *DIP*-chromosomal region and lacked strong support for the existence of a 'cold spot', a region in which recombination is suppressed, at the Mst78 and Mst53 loci located at ~3.5 cM from *DIP *at one flank.

**Figure 2:**
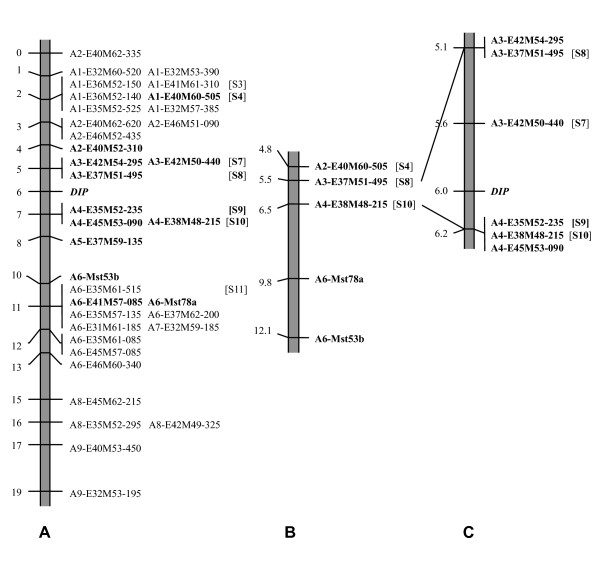
**Fine genetic linkage maps of *DIP *in *T. officinale***. **(A) **Map redrawn from Vijverberg *et al. *[38], based on 73 plants, 34 AFLP- and two Mst-markers, with minor updates (16, this study). The regular distribution of markers over the *DIP*-chromosomal region suggests the absence of suppression of recombination. **(B) **Map based on 300 plants and five PCR markers. The marker distribution is congruent with the absence of suppression of recombination and lacks evidence for the presence of a 'cold spot', a non-recombining region at the Mst loci. **(C) **Map based on the 24 recombinants between S8 and S10 out of 2227 plants tested and the six AFLPs known in this region. Markers closest to *DIP *are S7 at 0.4 cM at one flank and a cluster of three AFLPs, including S10, at 0.2 cM at the other flank, spanning a total of 0.6 cM. A1 to A9 denote AFLP-clusters according to manual sorting, and S3, S4, S7 to S11 denote sequence characterized regions.

An overview of the mapping results is given in Figure 2. Map A is redrawn from our previous study [38] with some minor updates [[16], this study]. It has a total length of 18.6 cM and shows a regular distribution of markers over the *DIP*-chromosomal region. *DIP *is located at one third of the map and markers co-segregating with *DIP *are lacking. Map B zooms in to a region of ~8 cM around *DIP*, based on 300 plants and five PCR markers. It confirms the occurrence of recombination in this region close to *DIP *and lacks strong evidence for local suppression of recombination at the Mst78-Mst53 region. Map C shows the fine-map of *DIP *based on the 24 recombinants between S8 and S10 out of the 2227 plants analyzed and the six most closely linked AFLPs. It shows partial resolution of the AFLP cluster at one flank of *DIP *and no further resolution of the AFLP cluster at the other flank of *DIP*. The length of the smallest *DIP*-chromosomal region was reduced to 0.6 cM, and the markers most closely linked to *DIP *are S7 and the cluster with S10 at 0.4 cM and 0.2 cM, respectively.

### Segregation distortion and incomplete penetrance of diplospory

**Figure 3:**
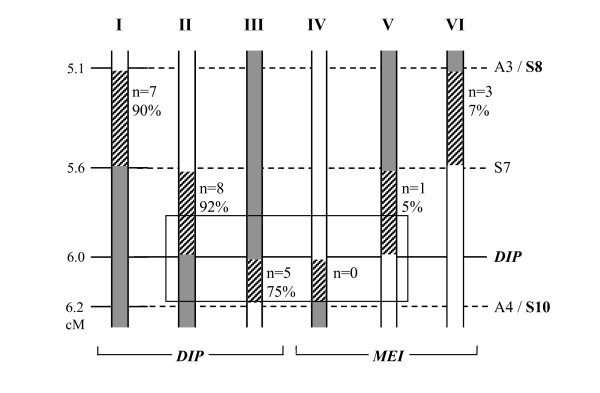
**Schematic representation of recombination types (I to VI), their frequency (n) and average penetrance of diplospory (%)**. The types reflect the different locations of recombination (stripes) and *DIP*-flank involved (dark grey bar), with white bars representing the non-*DIP *chromosomal parts. The schedule visualizes the bias towards diplosporous recombinants (20 out of 24) and indicates that this is irrespective of the *DIP*-flank involved. The boxed region suggests an enforcement of this bias closer to *DIP *(~all are diplosporous). The reduced average penetrance of diplospory found in recombinants of type III suggests that the region between *DIP *and S10 contains essential elements for diplosporous reproduction. Marker distances follow the fine-map of Figure 2C and penetrance of diplospory are further visualized in Figure 4.

In order to investigate whether the biased distribution towards diplosporous reproduction in the recombinants and the occurrence of a reduced penetrance of diplospory in some of them was correlated to a particular *DIP*-chromosomal region, the 24 recombinants were classified according to their place of recombination and *DIP*-flank involved. A total of six recombination types were possible, those between S8 and S7 (types I and VI; Figure 3), S7 and *DIP *(types II and V), and *DIP *and S10 (types III and IV), and their frequencies and accompanying average penetrance of diplospory were indicated (Figure 3). The scheme visualized the segregation distortion towards diplosporous recombinants (20 out of 24, see above) and showed that this was irrespective of the *DIP*-flank involved (types I *versus *VI, II *versus *V, and III *versus *IV). Incomplete penetrance of diplospory was associated with a region close to *DIP *at one flank (type III). Zooming in to the region between S7 and S10 showed an enforcement of the skewed distribution to 13 diplosporous *versus *one meiotic recombinant. Further zooming in to a region that covers ~0.2 cM at each side of *DIP *suggested that all recombinants are diplosporous here (Figure 3, boxed). Our results indicated that meiotic recombinants with a *DIP*-flank approaching *DIP *were either not formed or behaved diplosporously (type IV became included in type II and type V in type III, see discussion). They suggested that a reduced penetrance of diplospory is associated to a region close to *DIP*, particularly at the flank towards S10.

**Figure 4:**
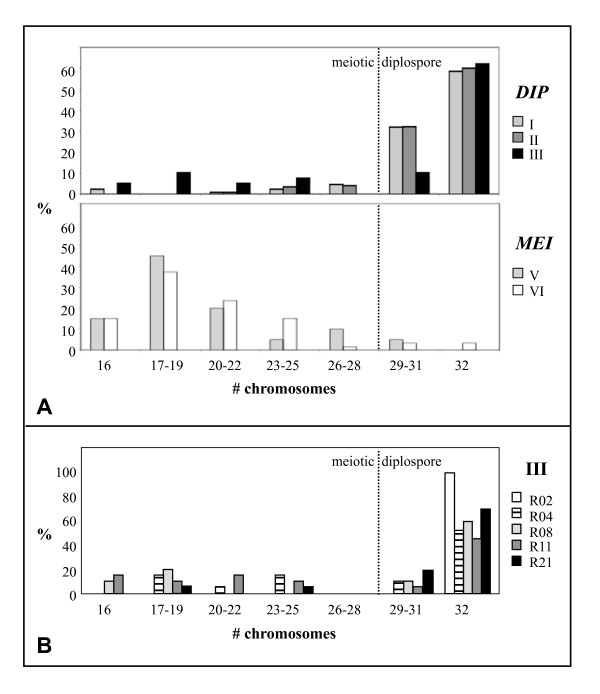
**Graphical representation of penetrance of diplospory**. The penetrance of diplospory is represented by the percentage of tetraploid plants among offspring of crosses between triploid recombinants and sexual diploid pollen donors. Chromosome numbers are derived from DNA contents as were determined by flow cytometry (see Methods). **(A) **Penetrance of diplospory averaged per recombination type (Figure 3), with diplosporous recombinants (n = 20) presented in the upper graph and meiotic ones (n = 4) in the lower graph. A minority of offspring plants, particularly of type III, showed incomplete penetrance of diplospory. **(B) **Penetrance of diplospory per plant of type III. Reduced penetrance were seen in plants R04 (stripes, 63%), R08 (grey, 80%), and R11 (dark grey, 50%), whereas R02 (white, 100%) and R21 (black, 90%) are normal diplosporous.

To further investigate the association of a reduced penetrance of diplospory to (a) particular chromosome region(s), the frequencies of offspring per chromosome number classes were depicted in a graph (Figure 4). This visualized the highest frequencies of reduced offspring in recombinants of type III (Figure 4A, black bars) and showed that these reductions were relatively severe, that is, they resulted in offspring with ≤ 24 chromosomes, indicating the formation of haploid to diploid eggs. Type III included the three plants with lowest penetrance of diplospory (50-70%), whereas the other three recombinants with reduced penetrance were of Types I and II (Figure 4A, grey bars). These plants showed less reduced penetrance of diplospory (75-80%) and also milder reductions, that is, they resulted in offspring with 24-32 chromosomes, indicating the formation of di- to triploid eggs. Recombinants of type III were depicted individually to further visualize the pattern of reduced penetrance of diplospory (Figure 4B). This showed strong reductions of diplospory in plants R04, R08 and R11 (stripes and grey bars) and normal penetrance in R02 and R21 (white and black bars). The summary of results suggested that diplosporous reproduction in *T. officinale *is regulated by a more complex locus rather than a single *DIP *gene (see discussion), with the region towards S10 particularly being important for full penetrance of diplospory.

### Comparative analysis between *DIP*- and meiotic homolog's

Sequence divergence between *DIP*- and non-*DIP *homolog's was investigated at six marker regions, representing both flanks and different distances to *DIP*, in order to further characterize the *DIP*-chromosomal region. For each region, PCR-products were obtained for the diploid sexual parent S2.125 (alleles *d*1 and *d*2), a triploid diplosporous offspring plant F1.15 (*D*, *d*1 and *d*3) and an unrelated natural apomictic A68 (*D*, *d*4 and *d*5) and sequences of cloned fragments analyzed. The results showed (many) more sequences than the expected two or three alleles per individual, indicating repetitivety of the *DIP*-linked sequences. As a result, *d *alleles could not always be identified unambiguously and two of the marker regions, S3 at ~4 cM at one flank of *DIP *and A4 from the cluster with S9 and S10 at ≥ 0.2 cM at the other flank of *DIP *were excluded. For the remaining four regions, sequence comparison was restricted to *d*1, *d*2 and *D*-alleles only, after defining them as follows: *d*1 and *d*2 were the most frequent and least diverged marker sequences in S2.125 of which *d*1 was also found in F1.15, and *D *was the sequence identical to the known *DIP-*sequence and present in the diplosporous F1.15 as well as apomictic A68. Results are given in Table 3, with '*p*' and '*q*' representing extra sequences that occurred more than once in an individual and '*singles*' indicating extra variants that were found only once. Sequence comparisons between *DIP *and non-*DIP *homolog's at S4 and S7 lacked significant differences (Table 3), indicating the absence of ASD at this flank at ≥ 0.4 cM from *DIP*. Sequence comparisons at S10-flank where more difficult to interpret, because the large divergence between *d*1 and *d2 *could possibly be the result of the inclusion of (a) paralog('s). The overrepresentation of the *D *allele in A68 may also point to sequence divergence at the primer sites of S10-flank at non-*DIP *homolog's. Since S10 is dominantly amplified (Figure 1), the presence of ASD at the S10 and possibly S10-flank region is indicated. For S11, the lack of *d*2 alleles and close similarity of *d*1 to *D *may indicate that sequence divergence between *d*1 and *d*2 is absent. No preferential amplification of *D *is indicated in F1.15, supporting a lack of ASD at this locus. In summary, our data lacks evidence for ASD at one flank > 0.4 cM from *DIP *and at the other flank at larger distances from *DIP*. They suggest possible ASD at the S10 and S10-flank region at ~0.2 cM from *DIP *at one flank.

**Table 3:** Sequence divergence at marker regions between the *DIP*-allele and its non-*DIP *homolog's in *T. officinale*

Marker region	S4						S7						S10-Flank						S11		
Distance to *DIP *(cM)	1.6						0.4						0.2						3.5		
Sequence length (bp)	375						317						329						323		
												
Plants	S	F	A^a^				S	F	A				S	F	A				S	F	A
							
Number of alleles																					
*d1*	7	5	-				5	2	-				5	1	-				15	8	-
*d2*	5	-	3				8	-	-				*2*	-	-				*1*	-	-
*D*	-	7	5				-	6	2				-	4	13				-	3	14
*p */*q *^b^	-	2	3				2/3	2	2/6				2	2/3	3				1	-	-
*singles*	5	1	5				2	4	7				1	4	2				-	6	1
Total	17	15	16				20	14	17				10	14	18				17	17	15
							
ASD (%)																					
*d1-d2*	1.6						2.8						*7.3 *^c^						0.6		
*d1-D*	0.8						2.2						3.0						1.5		
*d2-D*	1.9						3.9						*6.1*^c^						1.5		

^a ^S = S2.125; F = F1.15; A = A68

^b ^*p *and *q *represent sequences that were found more than once in an individual and possibly include the *d*3 allele in F1.15, *d*4 and *d*5 alleles in A68, or are paralogs; both *p *and *q *usually represent different sequences in the different individuals

^c ^this high percentage of ASD is probably the result of a comparison between paralogs rather than homolog's

## Discussion

### Recombination in the *DIPLOSPOROUS*-chromosomal region and the absence of overall allele sequence divergence make *DIP *a good candidate to isolate via a map-based cloning approach

Our data indicate that suppression of recombination plays a minor role in the *DIP*chromosomal region in *T. officinale*. This is supported by the equal distribution of markers over the *DIP*-map (Figure 2), the absence of markers co-segregating with diplospory, and the non-significantly deviating distances between the Mst78 and Mst53 alleles at *DIP *and non-*DIP *homolog's (Table 2 and Results). This result from the large segregating population confirms the conclusion of our initial mapping study [38], whereas possible local suppression of recombination at the Mst-region close to *DIP *is not supported. The occurrence of recombination in the *DIP*-chromosomal region is unique in comparison to other mapping studies of apomeiosis to date, that all showed strong suppression of recombination and in some cases hemizygosity at these loci (see Background) [reviewed by [16,25]]. This has frustrated map-based cloning efforts of genes associated to apomixis in the species concerned. In one of the best examples among them, FISH experiments indicated that the apospory-specific genomic region (ASGR) in *Pennisetum squamulatum *physically corresponds to ~50 Mbp or a quarter of a chromosome [54]. A recent mapping study in *P. squamulatum*, that made use of markers designed on long terminal repeat (LTR) sequences of ASGR-abundant transposon's, did also not resolve this region [55]. The occurrence of recombination around *DIP *in *T. officinale *offers a good opportunity to isolate an apomeiosis gene via further molecular marker searches based on the recombinants and BAC-walking from the markers most closely linked to *DIP*.

The present delimitation of *DIP *by S7 and S10 covers 0.6 cM. In comparison to tomato, which has a comparable genome size to *T. officinale *and for which 1 cM was found to correspond to 0.3-0.4 Mbp [56], the *DIP*-region is estimated to cover a physical length of ~200-300 Kb. With an average BAC-size of the *T. officinale *BAC-library of ~115 Kb [K Vijverberg *et al. *unpublished results] and supposing halfoverlapping BACs, this region could be closed by 4-6 BACs, making the isolation of *DIP *in *Taraxacum *feasible.

Our data show that overall ASD is lacking at the *DIP*-chromosomal region. This is supported by the occurrence of recombination (see former paragraph) and by comparative sequence data at three of the *DIP*-linked marker regions analyzed, S4, S7 and S11 (Figure 2; Table 3). At the fourth marker region, S10-flank, and also at S10, preferential amplification of the *DIP*-homolog was indicated. This, and the clustering of S10 with two other AFLPs, may be signs of ASD and hemizygosity at this region close to *DIP*, although, the severity and locality of these signs are yet unknown. Increased ASD in regions associated with asexual reproduction can be expected as a result of the accumulation of mutations in the absence of 'homogenization' with homolog's via recombination [39-41]. The strong suppression of recombination and/or hemizygosity found at most loci associated with apomixis [e.g., [29,54]] support this hypothesis. Also in sexuals, the occurrence of ASD in some regions of the genome has been indicated, e.g., haplotypic variability in maize was shown to strongly affect the frequency and distribution of recombination events [57]. Since *Taraxacum *is hermaphrodite, and apomixis concerns the female reproductive pathway only, recombination during pollen meiosis offers the possibility to retard ASD. By chance, overall this is expected to occur less frequently closer to *DIP*. Computer simulations of the depth of asexual reproduction history along a chromosome that carries a dominant single dose *DIP*-allele support this model [42]. It shows that the depth of asexual reproduction history accumulates with the number of generations also under sexual-asexual cycling conditions and is deepest at and close to *DIP*. This depth reduces at random generation times and positions across the flanks by incidental recombination events. When ASD becomes sufficiently pronounced, it will prevent for recombination also during pollen meiosis, which can lead to further increase of ASD. In addition to this gradual process, chromosome rearrangements may arise by chance and result in suppression of recombination immediately. In a heterozygous state, these mutations and rearrangements can accumulate without effect on apomictic seed production [45]. Our data are congruent with this model by suggesting that S4 and S7 at one flank of *DIP *and S11 at the other flank of *DIP *have been homogenized by relatively recent recombination events, and the region around S10, located closer to *DIP*, may have a deeper history of asexual reproduction. Since recombination in the *DIP*-region between S7 and S10 is still possible (Figure 3), the possible ASD at the S10-region may be explained by a local rearrangement.

An earlier study in *T. officinale *suggested that a certain level of ASD is associated with *DIP *[58,42], by showing severe segregation distortion of the Mst78a and Mst53b alleles that are linked to *DIP *in haploid pollen grains, but not in diploid ones. A plausible explanation for this is that the *DIP*-allele is not transferred via haploid pollen due to pollen lethality as a result of linked deleterious mutations [45]. These mutations would be masked by (the) non-*DIP *allele(s) in a diploid or polyploid stage. A study in *Tripsacum dactyloides *also suggested that genes for apomixis might be linked to loci that are subjected to segregation distortion in haploid but not diploid gametes [32]. When apomixis genes cannot be transferred via haploid pollen, this could also explain the prevalence of apomixis in polyploids [42]. Our data indicate that overall ASD is absent at the *DIP*-locus, but we argue that recombination during pollen meiosis may have resulted in a mosaic pattern of different depths of asexual reproduction history along the *DIP*-chromosome (see former paragraph). In addition, local rearrangements may have accumulated in regions associated with asexual reproduction. The linkage of *DIP *to a mutation load can then be explained by its confinement to (a) particular region(s) close to *DIP*. Alternatively, the non-transfer of *DIP *via haploid pollen is associated to the *DIP*-allele itself. A pleiotropic recessive pollen lethal effect linked to apospory in *Ranunculus auricomus *was suggested by Nogler [1]. Possibly, a similar pleiotropic effect has evolved in the *DIP*-gene or is associated to asexual reproduction in general, however, this needs further investigations.

**Figure 5:**
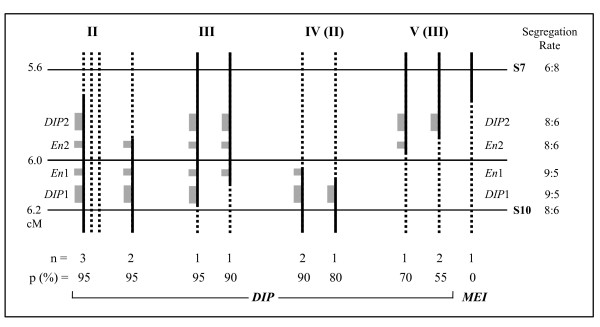
**One hypothetical composition of the *DIP*-locus in *T. officinale***. The scheme visualizes the recombination types between S7 and S10, concerning types II to IV from Figures 3 and 4, with further subdivision. The distances follow the fine-map of Figure 2C and the drawings follow Figure 1A. The most left type shows the non-*DIP *homolog's (dotted lines) in addition to the recombinant homolog (black line = *DIP*-part of the chromosome) of the triploid F1, and the other types show the S7/S10-recombinant homolog's only. Number of plants (n), penetrance of diplospory (p, in %), and reproduction types (*DIP*, *MEI*) are indicated. *DIP*1 and *DIP*2 (large grey boxes) represent the duplicated *DIP*-genes and *En*1 and *En*2 (smaller grey boxes) the enhancers or *cis*-regulators, with their segregation rates indicated. According to the results, *DIP*1 in combination with *En*1 results in full penetrance of diplospory, whereas one of the *DIP *genes alone or *DIP*2 in combination with *En*2 show reduced penetrance. Following this scheme, part of the recombinants of types II and III are supposed to actually belong to types IV and V, respectively. Alternative models tend to become more complex and are discussed in the text.

The occurrence of recombination and absence of overall ASD at the *DIP-*chromosomal region suggest that diplospory and apomixis are relatively recent in *T. officinale*. The clustering of markers found at the *PAR*-locus [PJ Van Dijk *et al. *unpublished results], however, seems to be in contrast to this conclusion. The evolution of either *DIP *or *PAR *alone is unlikely, since they lead to increased ploidy levels with each generation and haploid phenotypes, respectively, both expected to be evolutionary dead-ends in nature. The differences in marker clustering found at the *DIP *and *PAR *loci could possibly be explained by a different recombination history around these loci by chance [[42], see above], that has resulted in higher levels of ASD at the *PAR*- than *DIP*-locus. An alternative explanation is that the differences result from a difference in chromosome rearrangements at the *DIP*- and *PAR*-locus as compared to their non-*DIP *and non-*PAR *homolog's. Also differences in mitotic crossover and gene conversion may have played a role at these loci. Particularly one of these latter processes could have been involved in the origin of a duplication/inverted repeat in the *DIP*-locus as is postulated in Figure 5 (discussed below). The results possibly indicate that apomixis in *Taraxacum *is older than suggested by the *DIP*-chromosomal region alone.

In summary, our results suggest that ASD is absent in a wider region around *DIP*, non-pronounced close to *DIP*, and possibly present locally at the S10- and possible other regions linked to *DIP *as well as at the *PAR*-locus. This pattern of different levels of ASD linked to *DIP *and *PAR *is congruent with the outcome of computer simulations of recombination during pollen meiosis in regions associated with asexual reproduction and, in addition, different histories of local rearrangements. A practical implication of the absence of clear ASD at the *DIP*-locus is that non-*DIP *homolog's in the BAC-library can be used in the BAC-walking process in order to isolate *DIP*.

### Segregation distortion towards diplosporous recombinants and incomplete penetrance of diplospory in some of them indicate a duplicated *DIP*-locus

We found a five times over-representation of diplosporous plants among the 24 recombinants (Figure 3), whereas the 1:1 segregation rate of diplosporous *versus *meiotic reproduction was confirmed in a random set of 61 plants of the mapping population by phenotyping via crosses. This 1:1 segregation was also confirmed in the remainder of the 2227 plants analyzed, according to deduction from the S8/S10 genotypes (Table 1). The over-representation of diplosporous plants is thus indicated to be specific for plants with a recombination between S8 and S10. The bias towards diplosporous reproduction increases in recombinants with a *DIP*-flank closer to *DIP *at both flanks towards the presumed single *DIP *gene (Figure 3). The apparent segregation distortion could find its origin in one of two crosses: (i) the parental cross: S2.125 (2*x*) × PAX (4*x*, *D-allele*) (Figure 1A) or (ii) the crosses used for phenotyping: F1 (3*x*, including *D*/*d*-recombinants) × sexual (2*x*). In the parental cross, possible explanations for this bias are a preferential transfer of *DIP *to recombinant offspring plants or a decreased viability of meiotic recombinant F1's. In the crosses used for phenotyping, the bias is possibly explained by a shift towards diplosporous reproduction in recombinants with a *DIP*-flank approaching *DIP */the presence of a partial *DIP*-locus or by a misclassification of meiotic recombinants in diplosporous ones with reduced penetrance. In a tetraploid pollen donor, as is the case in the parental cross, normal bivalent formation and meiosis occur [e.g., [49]], making recombination events along the *DIP*-chromosome possible. In the diploid pollen as well as triploid offspring, a possible mutation load linked to *DIP*, as can be a result of long-term asexual reproduction, will be masked (see former paragraphs). If expressed, it will result in an under-representation of diplosporous individuals rather than an over-representation. If uncoupled, a mutation load could result in a reduced number of meiotic recombinants when it is selectively expressed in a sexual background. This does, however, not explain the reduced penetrance of *DIP *found in some of the recombinants and also not the effect found at both flanks: recombinants of both types IV and V are lacking (Figure 3). A modifier linked to *DIP *with a lethal effect in a sexual background, either in the pollen or early embryo stage, can be another possibility. Although uncoupling of the modifier may result in a lower expression of diplospory, also here, a single modifier does not explain the bias found at both flanks. The phenotyping of 61 random offspring plants also lacks signs of linkage of either favorable or deleterious mutations to diplospory. The segregation distortion towards diplosporous recombinants is, therefore, not strongly supported by a preferential transfer of diplospory or a decreased viability of meiotic offspring in the parental cross.

The segregation distortion of diplospory in the recombinants as well as the reduced penetrance of diplospory in some of them are better explained in the crosses used for phenotyping. Meiosis in these crosses occurs in triploid megasporocytes, making meiosis difficult and leading to high percentages of aneuploid gametes [e.g., [49]], whereas diplosporous non-reduction is unhindered. Although offspring plants resulting from aneuploid gametes survive (Figure 4), their number might be (slightly) reduced. The percentages of diplosporous offspring from plants with incomplete penetrance of diplospory may then, actually, be (slightly) lower. Accordingly, the six diplosporous recombinants with 50 to 85% penetrance of diplospory (Figures 3, 4), out of the total of 20, represent a minimum. In *Taraxacum*, unreduced, balanced triploid sperm cells can arise from a disturbed triploid true meiosis [e.g., [49,59], own observation], although at low frequencies not exceeding the 10%. Unreduced pollen formation is also known from other polyploidy species [60], and the few existing data for unreduced eggs suggest that the natural frequency of non-reduction is similar in megasporogenesis [[60] and references therein]. Occasional formation of triploid female gametes in meiotic triploid recombinants is, therefore, expected and indicated (Figure 4A, types V and VI). However, frequencies of ≥ 50% are too high to be explained without the action of diplospory. Moreover, segregation distortion and incomplete penetrance of diplospory was found in the recombinants only and not in the other diplosporous offspring of the mapping population. Apomixis in *Taraxacum *is usually obligate or close to obligate [48-50], also indicating full penetrance of diplospory under natural conditions. Together, this argues against misclassification of meiotic individuals as diplosporous ones with reduced penetrance. Our data is better explained by the presence of a relatively large *DIP*-locus in *Taraxacum *that, in part, still results in diplosporous reproduction and, depending on the parts available, could result in incomplete penetrance of diplospory.

A hypothetical constitution of the *DIP*-locus that explains the segregation distortion as well as incomplete penetrance of diplospory in some of the recombinants is given in Figure 5. Since the over-representation of the diplosporous phenotype is irrespective of the *DIP*-flank involved, a locus with a minimum of two *DIP*-genes is postulated. The absence of plants of recombination types IV and V (Figure 3) is then explained by their misclassification in types II and III, respectively, that is, the recombined allele excludes the putative single *DIP*-gene (Figure 5, at 6.0 cM), but still induces diplosporous reproduction. The different penetrance of diplospory is best explained by assuming one or two enhancers or *cis-*regulatory elements (Figure 5). Another possibility is additional *DIP*-genes that may result in copy number variation in the different recombinants (not shown), however, the number of copies increases to explain our data. A certain distance between the genes, and the enhancers/regulators, is supposed in order to give rise to the different recombinants within the proposed length of ≥ 200-300 Kb between S7 and S10. Since incomplete penetrance of diplospory is most pronounced in three plants of type III (Figures 3, 4), that have their recombination between *DIP *and S10, essential elements for obtaining diplospory are indicated to be located in this region. We postulate that a minimum requirement for obtaining full penetrance of diplospory is the presence of a complete *DIP*1 gene in combination with *En*1 (or *cis-*regulator1), whereas *DIP*1 or *DIP*2 alone or *DIP*2 in the presence of *En*2 only, result in reduced penetrance of diplospory.

Our results do not support a complex *DIP*-locus with different functional genes. Recently, the *osd*1/*Atrec*8/*Atspo*11-1 triple mutant in *Arabidopsis *was shown to produce a highly penetrant diplosporous phenotype: *mitosis *replaces *meiosis *(*MiMe*) [23]. In a similar way, the *DIP*-locus could consist of different functional genes. Genes with a combined action to produce a complex phenotype may be tightly linked as a result of co-evolution, e.g., like the self incompatibility loci in *Brassica *[61,62]. The overrepresentation of the diplosporous plants among the recombinants, however, argues against such a complex locus with different functional genes, since recombination would destroy the diplosporous phenotype.

Incomplete penetrance of apomeiosis was also found in *Erigeron annuus *[63] and *Poa pratensis *[35] and suggested to be correlated to a genetic background. In the study in *E. annuus*, a diplosporous triploid plant was pollinated by a sexual diploid, indicating that the genetic variation originated from the paternal plant and was not the result of maternal and epigenetic effects. The penetrance of diplospory was 92% in the triploid maternal parent and varied from 41 to 89% in the tetraploid offspring plants. Noyes [63] suggested either the presence of (*trans-*acting) modifiers that act directly on the expression of diplospory, novel epistatic interactions, or a combination thereof. The *Apospory initiator *(*Ait*) gene in *P. pratensis *[35] is a dominant gene needed for apospory and leading, together with four other genes, and depending on their differences in expressivity and interactions, to apomictic reproduction. *Ait *is of the aposporous type, not modifying meiosis, but depending on the formation of aposporous initials. The penetrance of *Ait *was found to vary between 0 and > 80%: it was absent in the maternal plant, but > 50% penetrant in some of the offspring originating either from selfing or from crossing with a sexual. Since incomplete penetrance of diplospory was found in some of the recombinants only and not in the diplosporous offspring plants without a recombination between S8 and S10, a genetic background that modifies the expression of *DIP *is not supported. Our results are better explained by a direct effect of recombination in the *DIP*-locus, as proposed in Figure 5, and not as a result of *trans*-acting effects.

Duplicated genes have often been associated with apomixis, but in the context of polyploidy [2] and not in the form of a tandem or inverted repeat at a single chromosome. Possible exceptions to this are the recently found duplicated *BABY BOOM *(*BBM*)-*like *gene and some duplicated putative protein-coding regions in the ASGR [20]. Hypothesized functional roles of ploidy include epigenetic gene deregulation, e.g., through changes in methylation [64], and asynchronous gene expression [65]. Others proposed that these complexities evolved upon establishment of apomictic reproduction and may be secondary [25]. In *Taraxacum *[58,42] and *Tripsacum *[32], segregation distortion of apomixis genes in haploid but not diploid gametes also suggest a secondary role of polyploidy. Whether a segmental duplication, as is postulated here, has a functional relationship to those hypothesized for a polyploid genome is yet unknown, but it is interesting to observe a possible gene dosage effect related to apomeiotic reproduction. Eventually map-based cloning of the *DIP*-locus will reveal the real structure of this locus and will give more insight into its function.

Diplospory results in FDR gametes, which are considered to be superior to 2*n-*gametes from second division restitution (SDR) in that they transfer all or a most of the parental heterozygosity and epistasis intact to their progeny. SDR gametes, on the other hand, result in near-homozygous offspring, varying in their chromosome constitution and possible heterozygosity only at the ends of the chromosome. Although both types allow for raising the ploidy level in artificial crosses, FDR gametes allow for pyramiding mutations or valuable characteristics in the same genetic background. *DIP *as well as the other apomixis related apomeiosis genes are dominant alleles (reviewed by 16). Whether they represent a new function or act via the inactivation of normal functions, i.e., deregulate the sexual pathway, is since long be debated [e.g., [3,64]). A few genes that showed deregulation of female meiosis in sexual model species, resulting in FDR-gametes, have been reported to date. These include *mob*1 in *Medicago sativa *[66,67], *dyad *in *Arabidopsis *[21,22], and a non-reduction mutant (*nrm*) in maize [64]. In contrast to *DIP*, these genes are all recessive. The mutants give the desired phenotype, but possibly via a different (de-)regulation than found in apomicts. Together with the useful applications of a dominant FDR-gene in plant breeding, isolation of *DIP *and finding out its function is desired and highly intriguing.

## Conclusions

In this study, we obtained interesting new insights into the structure of the locus for diplospory in *Taraxacum*. We fine-mapped *DIP *to a minimum of 0.6 cM, which is estimated to cover ~200-300 Kb, with the closest markers at 0.2 cM. We found a clear bias towards diplosporous recombinants in the *DIP*-region, irrespective of the *DIP-*flank involved. Some of the diplosporous recombinants showed incomplete penetrance of diplospory. We postulate a locus with a minimum of two *DIP*-genes, possibly including one or two enhancers or *cis-*regulatory elements, (Figure 5), on the basis of these results. Our data confirm the minor role of suppression of recombination and allele sequence divergence in the *DIP*-chromosomal region. This makes *DIP *an excellent candidate to isolate via a chromosome-walking approach. Mapping and isolating a gene that encodes unreduced female gamete formation and/or an element of apomixis is since long being undertaken. The progress in *Taraxacum *reported here is an important step closer to this aim.

## Methods

### Plant material

The mapping population of *T. officinale *used for initial mapping of *DIPLOSPOROUS *[38] was extended to a total of 2227 individuals. The population originated from a tetraploid diplosporous, but non-parthenogenetic, pollen donor (PAX, *2n *= *4x *= *32*) and a sexual diploid maternal parent (S2.125, *2n *= *2x *= *16*). PAX was the result of a cross between a non-apomictic, but diplosporous, triploid hybrid (H6-3 [51]) and a sexual diploid paternal plant. The tetraploid PAX produced sufficient diploid, reduced and recombined pollen to perform segregation studies on diplospory. The triploid offspring of PAX × S2.125 segregated c. 1:1 for diplosporous:meiotic reduction [44,38]. Seedlings were grown in the greenhouse and screened for recombination in the *DIP*-chromosomal region (described below). Recombinant plants were further raised in the greenhouse, phenotyped for diplospory, and analyzed for additional molecular markers in the *DIP*-chromosomal region.

### Screening for recombination in the *DIP*-chromosomal region

DNAs were screened for recombination between two PCR-markers that were derived from the dominant SCARs S8 and S10, spanning *DIP *in a total of 2.7 cM [38]. Total DNAs were isolated from c. 5 mg leaf tips via the 'RETCH method', using the CTAB procedure as described by Rogstad [68] and the modifications by Vijverberg *et al. *[38]. In this procedure, leaf tips were transferred to tubes (Costar 1.2 ml tubes 4408 and caps 4418) that contained two steel balls (bicycle, 1/8" in 60 gross, Tiangin, China) and were placed in racks (Costar) on ice. After harvesting, 500 μl CTAB-buffer was added to each tube, and leafs were homogenized via shaking the racks 4× 1 min. at 30 rps in a RETCH shaker (MM200, Düsseldorf, Germany). At the end of the isolation procedure, DNAs were resuspended in 100 μl T_0.1_E (10 mM Tris, 0.1 mM EDTA, pH = 8.0). Quality and quantity were checked by measuring the optical densities at 260 and 280 nm and analyzing 2 μl on 1% agarose gels.

PCRs were performed in 96-wells plates, using 2-10 ng DNA, 2.5 μl 10× PCRbuffer, 2.5 mM MgCl_2_, 0.5 mM dNTPs, 1% polyvinylpyrrolidone (PVP-40, Sigma- Aldrich), 0.2 mM of each primer, and 0.5 Units *Taq DNA Polymerase *(Expand High Fidelity PCR Systems, Roche Applied Science) in a total volume of 25 μl. One drop of mineral oil (Sigma-Aldrich) was used to overlay the reactions and PCRs were performed following 1× 2 min. 94°C, 38× (30 sec. 94°C, 30 sec. annealing temperature, and 1 min. 72°C), 1× 5 min. 72°C, and a cooling down to 12°C. Annealing temperatures of S8 and S10 primer pairs were 60°C and 57°C, respectively, and primer sequences are described in Vijverberg *et al. *[38]. PCR products were analyzed on 1.5% agarose gels, with diagnostic bands showing lengths of 446 bp and 168 bp respectively (Figure 1).

### *DIP*-phenotyping

Plants that showed recombination between S8 and S10 were phenotyped for diplosporous *versus *meiotic reduction via crosses, using sexual diploids as pollen donors [44]. The offspring was analyzed for their ploidy levels as deduced from flow cytometric analysis [59]. Diplosporous recombinants were expected to produce tetraploid offspring only, being the sum of a non-reduced triploid egg cell plus a haploid sperm cell [[51], type C plants]. They showed a DNA ratio = 2.0 (= 32 chromosomes) as compared to a diploid reference species. Meiotic recombinants produced a range of di-, tri-, and up to a few tetraploid offspring, representing the result of an aberrant meiotic reduction, typical for a triploid, plus a haploid male genome [[51], type A plants]. This was indicated by DNA ratios = 1.0 to 1.5 and some higher values. Ten offspring plants were analyzed of each of two crosses per recombinant. The penetrance of diplospory was calculated as the percentage of offspring out of the 20 plants measured that showed a DNA ratio ≥ 1.79 (≥ 29 chromosomes). This was after correction for the triploid recombinant parent by setting it's ratio to 1.5 (original ratios varied from 1.48 to 1.58). In natural triploids, the percentage of tetraploid offspring as a result of aberrant meiosis during microsporogenesis does not exceed 10% [[44], own observations]. As a 'wide' threshold, recombinants that showed < 30% tetraploid offspring in addition to reduced offspring were regarded meiotic, whereas the remainder was defined as diplosporous with a complete or reduced penetrance.

### Genetic fine-mapping of *DIP*

Plants that showed a recombination between S8 and S10 were analyzed for AFLPs known to be linked to *DIP *[[38], Figure 2A]. DNA was isolated from 0.5-0.6 g fresh leaves, using the CTAB protocol described by Rogstad [68], with the modifications suggested by Vijverberg *et al. *[38]. Isolated DNA was resuspended in 500 μl T_0.1_E, checked for content and purity as described above, and 200 ng was used for AFLP analyses. AFLPs were generated according to the protocol of Keygene N.V. (Wageningen, The Netherlands) version 2.2 described by Vos *et al. *[69], including the modifications described earlier [38]. AFLPs tested included those of the unresolved marker groups most closely linked to *DIP*: A3-E42M50-440 (= S7), A3-E37M51-495 (= S8), and A3-E42M54-295 at one flank of *DIP *and A4-E35M52-235 (= S9), A4-E38M48-215 (= S10), and A4-E45M53-090 at the other flank of *DIP*. Marker names represent the AFLP groups (A3, A4), enzymes (E = *Eco*RI, M = *Mse*I), selective nucleotides in alphabetical order (31 = AAA, 32 = AAC, 33 = AAG, etc.), and length of the fragments in base pairs. Seven markers at larger distances from *DIP *were analyzed as controls: A1-E40M60-505 (= S4), A2-E40M52-310, A2-E46M52- 435, A5-E37M59-135, A6-E41M57-85 and the microsatellite alleles A6-Mst78a and A6-Mst53b. AFLPs were analyzed on a *LI-COR IR*^2 ^automated sequencer (Biosciences, USA) and the relevant bands scored manually on the gel-output. Msts were analyzed on an *ALF *DNA sequencer (Pharmacia) and scored manually.

### Investigating the marker distribution over a wider *DIP*-chromosomal region

A subset of 300 individuals randomly chosen from the mapping population was, apart from S8 and S10, also screened for the polymorphic PCR markers S4, Mst78, and Mst53 that were located at larger distances from *DIP*, spanning a total of 9.6 cM. PCRs of S4 were performed as described for S8 and S10, using an annealing temperature of 53°C. Subsequently, 5 μl products were digested overnight at 55°C with 1 unit *Bse*GI (Fermentas GMBH, Germany) in 1× accompanying buffer in a reaction volume of 15 μl. Digests were analyzed on 1.5% agarose gels, showing undigested products of c. 430 bp and digested ones of 270 and 160 bp. The *DIP-*associated S4 products lacked the *Bse*GI recognition site, so that diplosporous individuals showed both, the undigested as well as digested products, whereas meiotic individuals showed digested products only. PCRs of Msts were performed as described by Falque *et al. *[43] and products were analyzed on a *LI-COR IR*^2 ^automated sequencer (Westburg, Leusden, The Netherlands). Since Mst-markers are co-dominant markers, with Mst78a and Mst53b being linked to *DIP*, they have the advantage to also resolve information about the meiotic homolog's involved.

### Data analysis and mapping

AFLP-, Mst-, and phenotypic data was collected in a binary data base in Excel. Linkage analyses were performed on datasets: A = the previous data set of 73 plants and 36 markers; B = the medium data set of 300 plants and five (to 36) markers; C = the entire data set of 2227 and two (to 36) markers. The data of each of the three data sets was sorted manually in such a way that recombination events were minimized. Linkage analyses were also performed by using Joinmap^® ^3.0 [70], with either all data or only the recombinants included. For the Joinmap analysis, the BC population type was used, with the Kosambi mapping function and threshold values for LOD > 1.0 and REC < 0.40. For some sets of recombinants, LOD and REC threshold values were relaxed to > 0.3 and < 0.45, respectively. An updated map A was drawn on the basis of the Joinmap output [[16], this study], confirming our previous results [38]. Maps B and C were drawn on the basis of the manual sortings and manual calculations of recombination frequencies. Since Joinmap analyses for these two data sets were inaccurate due to many missing values for most individuals, this was used as a guidance only. Confidence intervals of the map distances were calculated according to Wu *et al. *[71], regarding the chance that the true recombination fraction (*r*) is in the 95% interval under ± 1.96 √(*r*[1-*r*]/*n*) conditions, with *n *being the number of plants analyzed.

### Comparison of marker regions of *DIP*- *versus *non-*DIP *homolog's

Sequences were obtained for marker regions S3, S4, S7, S10-flank, A4-flank and S11, covering 300-400 bp regions located at different distances from *DIP *at both flanks (Figure 2A; Table 3). Individuals analyzed were the sexual diploid maternal parent S2.125, representing two meiotic homolog's (*d*1 and *d*2), a diplosporous triploid offspring plant, F1.15, representing the *DIP*-chromosome, one meiotic homolog originating from S2.125, and a second meiotic homolog (*D*, *d*1 and *d*3), and a natural triploid apomict, A68, containing the *DIP*-chromosome plus two meiotic homolog's (*D*, *d*4 and *d*5). PCR products were obtained as described above and in Vijverberg *et al. *[38], using as S7 reverse primer: 5'-TACTCACTTCCCGATCAACTCAC-3', and as S10-flank forward and reverse primers: 5'-TAGACTTGATGCTCCTTGACTTGG-3' and 5'-AAGGCTTCACATGAGGCAGTAAG-3', respectively. Products were cloned by using the pGem T Vector system II (Promega), according to the manuals description. Per individual, between 10 and 20 clones were sequenced per marker region (BaseClear, Leiden, The Netherlands), using an M13 primer. Sequences were compared by using the DNAstar software (Lasergene, London, UK) or CLC sequence viewer v6.2 (CLCbio, Aarhus, Denmark).

## Authors' contributions

KV participated in the design and coordination of the experiments, performed the genetic fine-mapping with AFLPs, comparative sequence analysis, and data and statistical analysis, and drafted and wrote the manuscript. SM carried out the RETCH DNA isolations and PCR-and Mst-marker screens. TB performed the *DIP-*phenotyping. PJvD conceived of the research, participated in the design and coordination of the experiments, and contributed to the discussion of the results and draft of the manuscript. All authors read and approved the final manuscript.

## References

[B1] Nogler hr GA Gametophytic apomixis: Embryology of angiosperms 1984 Springer- Verlag, Berlin

[B2] Bicknell RA Koltunow AM Understanding apomixis: Recent advances and remaining conundrums Plant Cell 2004 16 Suppl S228 S245 10.1105/tpc.017921 15131250 PMC2643386

[B3] Grimanelli D Leblanc O Perotti E Grossniklaus U Developmental genetics of gametophytic apomixis Trends Genet 2001 17 597 604 10.1016/S0168-9525(01)02454-4 11585667

[B4] Juel O Vergleichende untersuchungen über typische und parthenogenetische fortpflanzung bei der gattung *Antennaria* K Sven Vetenskapsakad Handl 1900 33 1 59

[B5] Juel O Die tetradenteilung bei *Taraxacum *und anderen Cichorieen K Sven Vetenskapsakad Handl 1906 39 1 21

[B6] Veilleux R Diploid and polyploid gametes in crop plants: Mechanisms of formation and utilization in plant breeding Plant Breed Rev 1985 3 253 288

[B7] Spillane C Steimer A Grossniklaus U Apomixis in agriculture: The quest for clonal seeds Sex Plant Reprod 2001 14 179 187 10.1007/s00497-001-0117-1 24573424

[B8] Vielle-Calzada JP Crane CF Stelly DM Apomixis: The asexual revolution Science 1996 274 1322 1323 10.1126/science.274.5291.1322

[B9] Miles JW Apomixis for cultivar development in tropical forage grasses Crop Sci 2007 47 Suppl S238 S249 10.2135/cropsci2007.04.0016IPBS

[B10] Kaushal P Agrawal A Malaviya DR Siddiqui SA Roy AK Ploidy manipulation in guinea grass (*Panicum maximum *Jacq., Poaceae) utilizing a hybridization-supplemented apomixis-components partitioning approach (HAPA) Plant Breed 2009 28 295 303 10.1111/j.1439-0523.2008.01567.x

[B11] Maynard-Smith J The evolution of sex 1978 Cambridge Univ Press, Cambridge, UK

[B12] Barton NH Charlesworth B Why sex and recombination? Science 1998 281 1986 1990 10.1126/science.281.5385.1986 9748151

[B13] Otto SP Lenormand T Resolving the paradox of sex and recombination Nat Rev Genet 2002 3 252 261 10.1038/nrg761 11967550

[B14] Hörandl E Grossniklaus U Van Dijk PJ Sharbel TF Apomixis: Evolution, mechanisms, and perspectives 2007 ARG Gantner Verlag, Rugell, Lichtenstein

[B15] Savidan Y Savidan Y, Carman JG, Dresselhaus T Transfer of apomixis through wide crosses The flowering of apomixis: From mechanisms to genetic engineering 2001 Mexico, D.F.: CIMMYT, IRD, European commission DG VI (FAIR) 153 167

[B16] Vijverberg K Van Dijk PJ Hörandl E, Grossniklaus U, Van Dijk PJ, Sharbel TF Genetic linkage mapping of apomixis loci Apomixis: Evolution, mechanisms, and perspectives 2007 ARG Gantner Verlag, Rugell, Lichtenstein 137 158

[B17] Curtis MD Grossniklaus U Molecular control of autonomous embryo and endosperm development Sex Plant Reprod 2008 21 79 88 10.1007/s00497-007-0061-9

[B18] Kaushal P Malaviya DR Roy AK Shalini Pathak Agrawal A Ambica Khare Siddiqui SA Reproductive pathways of seed development in apomictic guinea grass (*Panicum maximum *Jacq.) reveal uncoupling of apomixis components Euphytica 2008 164 81 92 10.1007/s10681-008-9650-4

[B19] Schallau A Arzenton F Johnston AJ Hähnel U Koszegi D Blattner FR Altschmied L Haberer G Barcaccia G Bäumlein H Identification and genetic analysis of the APOSPORY locus in *Hypericum perforatum *L Plant J 2010 62 773 784 10.1111/j.1365-313X.2010.04188.x 20202173

[B20] Conner JA Goel S Gunawan G Cordonnier-Pratt M-M Johnson VE Liang C W Haiming Pratt LH Mullet JE DeBarry J Yang L Bennetzen JL Klein PE Ozias-Akins P Sequence analysis of bacterial artificial chromosome clones from the apospory-specific genomic region of *Pennisetum *and *Cenchrus* Plant Physiol 2008 147 1396 1411 10.1104/pp.108.119081 18508959 PMC2442526

[B21] Mercier R Vezon D Bullier E Motamayor JC Sellier A Lefèvre F Pelletier G Horlow C *SWITCH*1 (*SWI*1): A novel protein required for the establishment of sister chromatid cohesion and for bivalent formation at meiosis Genes Dev 2001 15 1859 71 10.1101/gad.203201 11459834 PMC312743

[B22] Ravi M Marimuthu MP Siddiqi I Gamete formation without meiosis in *Arabidopsis* Nature 2008 451 1121 1124 10.1038/nature06557 18272967

[B23] d'Erfurth I Jolivet S Froger N Catrice O Novatchkova M Mercier R Turning meiosis into mitosis PLoS Biol 2009 7 e1000124 10.1371/journal.pbio.1000124 19513101 PMC2685454

[B24] Olmedo-Monfil V Durán-Figueroa N Arteaga-Vázquez M Demesa-Arévalo E Autran D Grimanelli D Slotkin RK Martienssen RA Vielle-Calzada J-P Control of female gamete formation by a small RNA pathway in *Arabidopsis* Nature 2010 464 628 632 10.1038/nature08828 20208518 PMC4613780

[B25] Ozias-Akin P Van Dijk PJ Mendelian genetics of apomixis in plants Annu Rev Genet 2007 41 509 537 10.1146/annurev.genet.40.110405.090511 18076331

[B26] Ebina M Nakagawa H Yamamoto T Araya H Tsuruta S Takahara M Nakajima K Co-segregation of AFLP and RAPD markers to apospory in Guinea grass *Panicum maximum *(Jacq) Grassl Sci 2005 51 71 78 10.1111/j.1744-697X.2005.00011.x

[B27] Pupilli F Labombarda P Caceres ME Quarín CL Arcioni S The chromosome segment related to apomixis in *Paspalum simplex *is homoeologous to the telomeric region of the long arm of rice chromosome 12 Mol Breed 2001 8 53 61 10.1023/A:1011966922301

[B28] Labombarda P Busti A Caceres ME Pupilli F Arcioni S An AFLP marker tightly linked to apomixis reveals hemizygosity in a portion of the apomixis-controlling locus in *Paspalum simplex* Genome 2002 45 513 519 10.1139/g02-014 12033620

[B29] Ozias-Akins P Roche D Hanna WW Tight clustering and hemizygosity of apomixis-linked molecular markers in *Pennisetum squamulatum *implies genetic control of apospory by a divergent locus that may have no allelic form in sexual genotypes Proc Natl Acad Sci USA 1998 95 5127 5132 10.1073/pnas.95.9.5127 9560240 PMC20225

[B30] Jessup RW Burson BL Burow GB Wang Y-W Chang C Li Z Paterson AH Hussey MA Disomic inheritance, suppressed recombination, and allelic interactions govern apospory in buffelgrass as revealed by genome mapping Crop Sci 2002 42 1688 1694 10.2135/cropsci2002.1688

[B31] Goel S Chen Z Akiyama Y Conner JA Basu M Gualtieri G Hanna WW Ozias-Akins P Comparative physical mapping of the apospory-specific genomic region in two apomictic grasses Pennisetum squamulatum and *Cenchrus ciliaris* Genetics 2006 173 389 400 10.1534/genetics.105.054429 16547108 PMC1461418

[B32] Grimanelli D Leblanc O Espinosa E Perotti E González-de-León D Savidan Y Mapping diplosporous apomixis in tetraploid *Tripsacum*: One gene or several genes? Heredity 1998 80 33 39 10.1046/j.1365-2540.1998.00263.x 9474774

[B33] Pessino SC Evans C Ortiz JPA Armstead I do Valle CB Hayward MD A genetic map of the apospory-region in *Brachiaria *hybrids: Identification of two markers closely associated with the trait Hereditas 1998 128 153 158 10.1111/j.1601-5223.1998.00153.x

[B34] Barcaccia G Mazzucato A Albertini E Zethof J Gerats A Pezzotti M Falcinelli M Inheritance of parthenogenesis in *Poa pratensis *L: Auxin test and AFLP linkage analyses support monogenic control Theor Appl Genet 1998 97 74 82 10.1007/s001220050868

[B35] Matzk F Prodanovic S Bäumlein H Schubert I The inheritance of apomixis in *Poa pratensis *confirms a five locus model with differences in gene expressivity and penetrance Plant Cell 2005 17 13 24 10.1105/tpc.104.027359 15608334 PMC544486

[B36] Noyes RD Rieseberg LH Two independent loci control agamospermy (apomixis) in the triploid flowering plant *Erigeron annuus* Genetics 2000 155 379 390 10790411 10.1093/genetics/155.1.379 PMC1461076

[B37] Catanach AS Erasmuson SK Podivinsky E Jordan BR Bicknell R Deletion mapping of genetic regions associated with apomixis in *Hieracium* Proc Natl Acad Sci USA 2006 103 18650 18655 10.1073/pnas.0605588103 17047034 PMC1693717

[B38] Vijverberg K Van Der Hulst RG Lindhout P Van Dijk PJ A genetic linkage map of the diplosporous chromosomal region in *Taraxacum officinale *(common dandelion; Asteraceae) Theor Appl Genet 2004 108 725 732 10.1007/s00122-003-1474-y 14564398

[B39] Birky CW Jr Heterozygosity, heteromorphy, and phylogenetic trees in asexual eukaryotes Genetics 1996 144 427 437 8878706 10.1093/genetics/144.1.427 PMC1207515

[B40] Mark Welch DB Mark Welch JL Meselson M Evidence for degenerate tetraploidy in *Bdelloid rotifers* Proc Natl Acad Sci USA 2008 105 5145 5149 10.1073/pnas.0800972105 18362354 PMC2278229

[B41] Mark Welch D Meselson M Evidence for the evolution of *Bdelloid rotifers *without sexual reproduction or genetic exchange Science 2000 288 1211 1215 10.1126/science.288.5469.1211 10817991

[B42] Van Dijk PJ De Jong H Vijverberg K Biere A Schön I, Martens K, Van Dijk PJ An apomixis-gene's view on dandelions Lost sex: The evolutionary biology of parthenogenesis 2009 Springer, London, UK 475 495 full_text

[B43] Falque M Keurentjes J Bakx-Schotman JMT Van Dijk PJ Development and characterization of microsatellite markers in the sexual-apomictic complex *Taraxacum officinale *(dandelion) Theor Appl Genet 1998 97 283 292 10.1007/s001220050897

[B44] Van Dijk PJ Bakx-Schotman T Formation of unreduced megaspores (diplospory) in apomictic dandelions (*Taraxacum officinale*, *s.l*.) is controlled by a sex-specific dominant locus Genetics 2004 166 483 492 10.1534/genetics.166.1.483 15020437 PMC1470670

[B45] Richards AJ Agamospermy Plant breeding systems 1997 Chapman and Hall, London, UK 396 450

[B46] Gustafsson Å Primary and secondary association in *Taraxacum* Hereditas 1934 20 1 31 10.1111/j.1601-5223.1935.tb03176.x

[B47] Gustafsson Å Apomixis in higher plants III: Biotype and species formation Lunds Univ Arrskr 1947 43 183 370

[B48] Malecka J Embryological studies in *Taraxacum palustre* Acta Biol Crac Ser Bot 1965 8 223 235

[B49] Malecka J Problems of the mode of reproduction in microspecies of *Taraxacum *section *Palustria *Dahlsted Acta Biol Crac ser Bot 1973 16 37 84

[B50] Van Baarlen P Van Dijk PJ Hoekstra RF De Jong JH Meiotic recombination in sexual diploid and apomictic triploid dandelions *Taraxacum officinale *(L) Genome 2000 43 827 835 10.1139/gen-43-5-827 11081973

[B51] Van Dijk PJ Tas ICQ Falque M Bakx-Schotman T Crosses between sexual and apomictic dandelions (*Taraxacum*) II: The breakdown of apomixis Heredity 1999 83 715 721 10.1038/sj.hdy.6886200 10651916

[B52] Sørensen T Sexual chromosome-aberrants in triploid apomictic *Taraxaca* Bot Tidskr 1958 54 1 22

[B53] Fürnkranz D Cytogenetischen untersuchungen an *Taraxacum *im raume von Wien II: Hybriden zwischen *T. officinale *und *T. pallustre* Oesterr Bot Zeitschift 1961 108 408 415 10.1007/BF01289747

[B54] Akiyama Y Hanna WW High-resolution physical mapping reveals that the apospory-specific genomic region (ASGR) in *Cenchrus ciliaris *is located on a heterochromatic and hemizygous region of a single chromosome Theor Appl Genet 2005 111 1042 1051 10.1007/s00122-005-0020-5 16133318

[B55] Huo H Conner JA Ozias-Akins P Genetic mapping of the apospory-specific genomic region in *Pennisetum squamulatum *using retrotransposon-based molecular markers Theor Appl Genet 2009 119 199 212 10.1007/s00122-009-1029-y 19370319

[B56] Chang S-B Anderson LK Sherman JD Royer SM Stack SM Predicting and testing physical locations of genetically mapped loci on tomato pachytene chromosome Genetics 2007 176 2131 2138 10.1534/genetics.107.074138 17565940 PMC1950619

[B57] He L Dooner HK Haplotype structure strongly affects recombination in a maize genetic interval polymorphic for *Helitron *and retrotransposon insertions Proc Natl Acad Sci USA 2009 106 8410 8416 10.1073/pnas.0902972106 19416860 PMC2688972

[B58] Van Dijk PJ Ecological and evolutionary opportunities of apomixis: Insights from *Taraxacum *and *Chondrilla* Philos Trans R Soc Lond B Biol Sci 2003 35 1113 1121 10.1098/rstb.2003.1302 PMC1693208 12831477

[B59] Tas ICQ Van Dijk PJ Crosses between sexual and apomictic dandelions (*Taraxacum*) I: The inheritance of apomixis Heredity 1999 83 707 714 10.1038/sj.hdy.6886190 10651915

[B60] Ramsey J Schemske DW Pathways, mechanisms, and rates of polyploidy formation in flowering plants Annu Rev Ecol Syst 1998 29 467 501 10.1146/annurev.ecolsys.29.1.467

[B61] Shiba H Kenmochi M Sugihara M Iwano M Kawasaki S Suzuki G Watanabe M Isogai A Takayama S Genomic organization of the S-locus region of *Brassica* Biosci Biotechnol Biochem 2003 67 622 626 10.1271/bbb.67.622 12723613

[B62] Takuno S Fujimoto R Sugimura T Sato K Okamoto S Zhang SL Nishio T Effects of recombination on hitchhiking diversity in the *Brassica *selfincompatibility locus complex Genetics 2007 177 949 958 10.1534/genetics.107.073825 17720932 PMC2034657

[B63] Noyes RD Inheritance of apomeiosis (diplospory) in fleabanes (*Erigeron*, Asteraceae) Heredity 2005 94 193 198 10.1038/sj.hdy.6800597 15536486

[B64] Curtis MD Grossniklaus U Hörandl E, Grossniklaus U, Van Dijk PJ, Sharbel TF Amphimixis and apomixis: Two sides of the same coin! Apomixis: Evolution, mechanisms, and perspectives 2007 ARG Gantner Verlag, Rugell, Lichtenstein 37 62

[B65] Carman JG Hörandl E, Grossniklaus U, Van Dijk PJ, Sharbel TF Do duplicate genes cause apomixis? Apomixis: Evolution, mechanisms, and perspectives 2007 ARG Gantner Verlag, Rugell, Lichtenstein 63 92

[B66] Barcaccia G Albertini E Rosellini D Tavoletti S Veronesi F Inheritance and mapping of 2*n*-egg production in diploid alfalfa Genome 2000 43 528 537 10.1139/gen-43-3-528 10902718

[B67] Citterio S Albertini E Varotto S Feltrin E Soattin M Marconi G Sgorbati S Lucchin M Barcaccia G Alfalfa *Mob*1-like genes are expressed in reproductive organs during meiosis and gametogenesis Plant Mol Biol 2005 58 789 807 10.1007/s11103-005-8104-9 16240174

[B68] Rogstad SH Saturated NaCl-CTAB solution as a means of field preservation of leaves for DNA analyses Taxon 1992 41 701 708 10.2307/1222395

[B69] Vos P Hogers R Bleeker M Reijans M Vandelee T Hornes M Frijters A Pot J Peleman J Kuiper M Zabeau M AFLP: A new technique for DNAfingerprinting Nucleic Acids Res 1995 23 4407 4414 10.1093/nar/23.21.4407 7501463 PMC307397

[B70] Van Ooijen JW Voorrips RE Joinmap^® ^3.0. Software for the calculation of genetic linkage maps Plant Res Int (PRI) 2001 Wageningen, The Netherlands

[B71] Wu KK Burnquist W Sorrells ME Tew TL Moore PH Tanksley SD The detection and estimation of linkage in polyploids using single-dose restriction fragments Theor Appl Genet 1992 83 294 300 10.1007/BF00224274 24202510

